# Potential Mechanisms of Dietary Potassium Diformate and Sodium Propionate Driving Intestinal Microbiota and Lipid Metabolites to Modulate Intestinal Health of *Trachinotus ovatus*

**DOI:** 10.1155/anu/5594216

**Published:** 2025-11-18

**Authors:** Pengwei Xun, Qianqian Huang, Heizhao Lin, Dexiang Feng, Shengzhe An, Yujie Lei, Yuanye Ma, Chuanpeng Zhou, Jiahui Liu, Wei Yu

**Affiliations:** ^1^School of Fisheries, Xinyang Agriculture and Forestry University, Xinyang 464000, China; ^2^Key Laboratory of South China Sea Fishery Resources Exploitation and Utilization, Ministry of Agriculture and Rural Affairs; South China Sea Fisheries Research Institute, Chinese Academy of Fishery Sciences, Guangzhou 510300, China; ^3^Shenzhen Base of South China Sea Fisheries Research Institute, Chinese Academy of Fishery Sciences, Shenzhen 518121, China; ^4^Xinyang Nanwan Reservior Fishery Development Co., Ltd., Xinyang 464000, China

**Keywords:** gut health, lipid profile, potassium diformate, sodium propionate, *Trachinotus ovatus*

## Abstract

This study aims to investigate the effects of potassium diformate (KDF) and sodium propionate (NaP) on gut digestive and immune functions, intestinal microbiota, and lipid metabolites of *Trachinotus ovatus* based on multiomics approach. Juvenile *T. ovatus* (initial weight: 8.65 ± 0.02 g) were subjected to a 56-day feeding regimen. Three isonitrogenous and isolipidic diets, including the control (CG), an additional 6.6 g/kg of KDF, and an additional 6.0 g/kg of NaP were fed to juvenile fish twice daily. Results showed that KDF and NaP supplementation significantly increased the activities of chymotrypsin (Chy), lipase (Lip), α-amylase (α-amy), creatine kinase (CK), Na^+^K^+^-ATPase (Na^+^K^+^-ATP), γ-glutamyl transferase (γ-GT), alkaline phosphatase (AKP), glutathione peroxidase (GSH-Px), total antioxidant capacity (T-AOC), and superoxide dismutase (SOD) as well as the expression level of *Nrf2* (*p* < 0.05), while decreased the pH value, malondialdehyde (MDA) content and the mRNA level of *Keap1* (*p* < 0.05). Dietary KDF and NaP markedly enhanced microbial α-diversity and induced significant shifts in microbiota composition through selective modulation of bacterial populations, such as *Photobacterium*, *Mycoplasma*, and *Mycobacterium* (*p* < 0.05). Besides, KDF and NaP led to notable alterations in the intestinal metabolite lipidome through increasing short-chain fatty acids (SCFAs) levels, upregulating the abundance of phosphatidylcholine (PC), phosphatidylethanolamine, methyl PC (MePC), lysophosphatidic acid, ceramide (Cer), sitosteryl, monogalactosyldiacylglycerol, coenzyme, and lysophosphatidylethanolamine and downregulating the abundance of sphingomyelin and monoglyceride (*p* < 0.05). The assessment of associations revealed inverse relationships of digestive and antioxidative indices with *Photobacterium*, but positive correlations with *Mycoplasma*, *Mycobacterium*, *Ruegeria*, *Synechococcus*, *Nautella*, *Turicibacter*, and *Roseovarius*. This study advances our understanding of KDF and NaP on intestinal health.

## 1. Introduction

Growing requirements on aquatic farming yields has catalyzed the implementation of intensified cultivation frameworks. However, the intensive aquaculture predisposed to damage intestine of fish resulting in an increased risk of reduction of immunity, imbalances of nutrition, and eruption of infectious diseases [1]. Gut health is influenced by host, dietary nutrients, environmental factors, and microbes [2]. There is growing evidence that gut microbiota has emerged as a critical factor in the maintenance and regulation of intestinal homeostasis [3]. Gut bacteria can increase dietary nutrient bioavailability via secretory enzyme synthesis [4]. Moreover, gut bacteria can interact with host natural killer (NK) cells, neutrophils, and monocytes and induce the expression of intestinal cytokines to modulate the innate immune system of fish for certain immunological benefits [5, 6]. Besides, the metabolites derived by gut microbiota could regulate host metabolic health by producing and transforming a range of metabolites and molecules, such as short-chain fatty acids (SCFAs), bile acids, endocannabinoids, and bioactive lipids [3, 7]. Studies have confirmed that an effective strategy for improving the intestinal health is to use some safe and bioactive compounds that stimulate probiotic activity and digestive enzyme production while suppressing pathogens [8–11].

SCFAs refer to fatty acids with fewer than 6 carbon atoms, such as formic acid, acetic acid, and propionic acid. As a type of promising functional additive, SCFAs have played important roles in aquaculture [12]. Potassium diformate (KDF) and sodium propionate (NaP) are stabilized conjugate salts of formic acid and propionic acid, respectively, which can affect lipid metabolism and regulate immune system of host [13]. It has been established in extensive research that KDF and NaP can enhance the growth of various fishery animals [14–20]. KDF and NaP also play regulatory roles in gut health of fish. For instance, inclusion of KDF in the diet improved the gut morphology of Nile tilapia, (*Oreochromis niloticus*) [21], sterlet sturgeon (*Acipenser ruthenus*) [22], and rohu (*Labeo rohita*) [23], and ameliorated intestinal microbiota of hybrid tilapia (*Oreochromis niloticus* female × *O. aureus* male) [24] and grass carp (*Ctenopharyngodon idella*) [25]. Dietary NaP improved digestive enzyme activities, gut microbiota, and disease resistance in yellowfin seabream (*Acanthopagrus latus*), crucian carp (*Carassius carassius*), European seabass (*Dicentrarchus labrax*), and red drum (*Sciaenops ocellatus*) [26–29]. However, current research concerning the effects of KDF and NaP on gut health mainly focuses on the analysis of intestinal morphology, immunobiochemical markers and gut microbiota composition, while studies elucidating on gut metabolomic profiles and the correlation analysis integrating gut microbiota, physiological indicators and metabolic products is scarce.

Recent advances in omics-based high-throughput screening platforms have established their efficacy in pathogenesis modeling and biomarker validation. Lipidomics focuses on characterizing the structural diversity, quantitative profiles, and biological roles of lipid molecules within metabolic pathways [30, 31]. Alterations in the lipidome of intestinal content often serve as early biochemical hallmarks of functional alterations in the gut [32, 33]. As a result, identification of alterations to lipid profiles within the intestinal tract of fish has gained significant attention in the investigation of gut health.

Golden pompano (*Trachinotus ovatus*) is a cornerstone species in China. Our previous studies have confirmed dietary KDF and NaP can improve the growth, immunological resistance, and intestinal histology of *T. ovatus* [34, 35]; however, their impact on intestinal microbiota and gut lipid profiles of *T. ovatus* remains unexplored. Consequently, this research aimed to examine the impact of dietary KDF and NaP on intestinal health of juvenile *T. ovatus* by analyzing the intestinal digestive and antioxidative functions, SCFAs contents, microorganism composition, and lipidomic profiles. This study will provide novel insights into the role of SCFAs in facilitating fish intestinal health.

## 2. Materials and Methods

### 2.1. Experimental Diets

Three dietary treatments were established: a basal diet (CG), a diet enriched with 6.6 g/kg KDF (purity ≥98%, Changyi Pharmaceutical Co., Ltd.), and a diet enriched with 6.0 g/kg NaP (purity ≥99.0%, Shanghai Sangon Biotech Co., Ltd.) (Table S1). The optimal KDF and NaP level were obtained from our prior studies [34, 35]. Powdering of all ingredients was carried out followed by sieving through a 425-μm sieve. Subsequently, fish oil was combined with the aqueous phase that had been integrated into the mixture. Next pelletize the mixture into pellets (2.0 and 2.5 mm) by a pelletizer. After preparation, the diets were dried in air for 24 h and preserved at −20°C until needed for experimentation.

### 2.2. Animal Experiments

A controlled nutritional experiment was carried out in a pond system with seawater conditions located at the CAFS-affiliated Shenzhen Base (Chinese Academy of Fishery Sciences). *T. ovatus* juveniles were temporarily reared for 14 days using the CG diets for acclimating to experimental conditions. Random assignment partitioned 225 healthy fish (8.65 ± 0.02 g mean weight) among nine net cages (1.8 m^3^). Three replicate cages constituted each treatment, maintaining 25 individuals per enclosure. Fish were fed to satiation using the respective diets twice daily (6:30 and 17:30) for 8 weeks. Critical variables of aquaculture water were summarized hereafter: temperature 30.83 ± 0.43°C, pH 7.77 ± 0.10, dissolved oxygen 5.92 ± 0.11 mg/L, salinity 17.42 ± 0.19%, dissolved ammonia < 0.05 mg/L, and nitrite <0.01 mg/L.

### 2.3. Sampling

All of fish were maintained in a fasted state 6 h after the last feeding. Five fish were randomly selected from each cage, anesthetized with 100 mg/L eugenol, and sacrificed. Then the intestines were excised aseptically and subjected to the analysis of pH, digestive, and antioxidative ability. Another five fish per cage were sacrificed randomly and the intestinal contents were gathered, cryopreserved in liquid nitrogen, and maintained at −80°C pending analytical procedures of SCFAs levels, microbiota and lipidomics. Considering individual differences, gut contents of three parallel groups were blended, and respectively, analyzed SCFA levels, microbiota, and lipid profiles. In this way, each sample mixed intestinal contents from 15 fish.

### 2.4. pH and Enzymatic Activities in the Intestine

The intestinal digesta were homogenized in deionized water (1:9, w/v) for 1 min with a portable homogenizer. Then pH was measured by a pH measurement device (SMART SENSOR, Dongguan, China) according to the method of Baruah et al. [36]. Intestine samples were weighed and subsequently homogenized with ice-cold physiological saline (1:9, tissue: saline). The resulting supernatant was used to determine the activities of chymotrypsin (Chy, A080-3-1), lipase (Lip, A054-2-1), α-amylase (α-amy, C016-1-2), creatine kinase (CK, BC1145), Na^+^K^+^-ATPase (Na^+^K^+^-ATP, BC0065), γ-glutamyl transferase (γ-GT, BC1225), alkaline phosphatase (AKP, A059-2-2), glutathione peroxidase (GSH-Px, A005-1-2), total antioxidant capacity (T-AOC, A015-2-1), superoxide dismutase (SOD, A001-3-2), and malondialdehyde (MDA, A003-1-2) as described by the manual of commercial kits using the Absorbance Microplate Reader (VersaMax, SMP500-18170-OUJL, USA).

### 2.5. Gene Expression Related to the *Keap1* and *Nrf2*

Total RNA of the intestine tissue was obtained via TRIZOL extraction. The RNA yield and integrity, cDNA synthesis and the comparative transcriptional abundance of the target genes were processed using established protocols [34]. The specific information about the target genes, including primer sequences, amplified product lengths, and annealing temperature, is presented in the supplementary method (Table S2). β-Actin served as the reference gene. Target genes abundance was normalized to the reference gene and analyzed via the method of Livak and Schmittgen [37].

### 2.6. Intestinal Microbiota

The intestinal microbiota was measured using the 16S rRNA sequencing technique in Guangzhou Jirui Gene Technology Co., Ltd. The gut microbiota profiling was assessed followed the methodology from our prior studies [33]. Briefly, the total DNA of the bacteria was extracted and then amplified and sequenced after the detection of DNA quality. The V3–V4 regions of 16S rRNA were selected for the amplification and data analysis were constructed on the company platform.

### 2.7. SCFA Levels of Intestinal Contents

Intestinal SCFAs levels were tested by gas chromatography–mass spectrometry according to our previous study [33]. In short, the intestinal contents were homogenized in 15% phosphate buffer containing isocaproic acid (internal standard, 125 μg/mL) and ethyl ether for 1 min. Then the homogeneity was centrifuged at 12,000 r/min for 10 min at 4°C to obtain the supernatant. Finally, the sample were tested on the machine.

### 2.8. Lipidome of Intestinal Contents

Lipid were extracted from intestinal contents using the methodology described by Xun et al. [33]. Chromatographic separation was carried out on a Thermo (Vanquish) liquid chromatography system, which was equipped with a ACQUITY UPLC BEH C18 column (2.1 mm × 100 mm, 1.7 μm particle size). Mass spectrometry was performed in a Thermo (QE) mass spectrometer. A comprehensive lipid annotation was performed on the acquired raw data using LipidSearch software (v4.0). The annotation results were then subjected to alignment and sum peak normalization using LipidSearch software. Following data preprocessing, the annotated lipids were categorized based on their fatty acyl chains and head groups. Multivariate statistical analysis, differential lipid screening, and analysis were performed using the roplspackage in R, with visualization conducted via the OmicStudio online tools (https://www.omicstudio.cn).

### 2.9. Statistical Analysis

The variance homogeneity and normality of data for all physiological indicators, SCFAs concentration, alpha diversity indices, and microbiota abundance were tested by the Shapiro–Wilk test and Levene's test procedures of SPSS 23.0 (SPSS, Michigan Avenue, Chicago, IL, USA), respectively. Statistical analysis was performed using one-way ANOVA to assess differences among groups. When a significant effect was found, statistical comparisons of means were carried out using Tukey's test, with a significance level of *p* < 0.05. The statistical model used for the ANOVA is expressed as follows:

  $$\begin{array}{c} Y=µ+\alpha +\varepsilon , \end{array}$$

where *μ* signifies the grand mean, *α* represents treatment effects attributable to group differences, and *ε* constitutes the stochastic disturbance. Results are given as means ± standard error means (SEM) and the corresponding bar diagrams were prepared with Graph Pad Prism 8.0. Data visualization and interpretation of omics-related figures were conducted on the OmicStudio cloud platform (https://www.omicstudio.cn/tool).

## 3. Results

### 3.1. Intestinal pH and Biochemical Indicators

**Figure 1:**
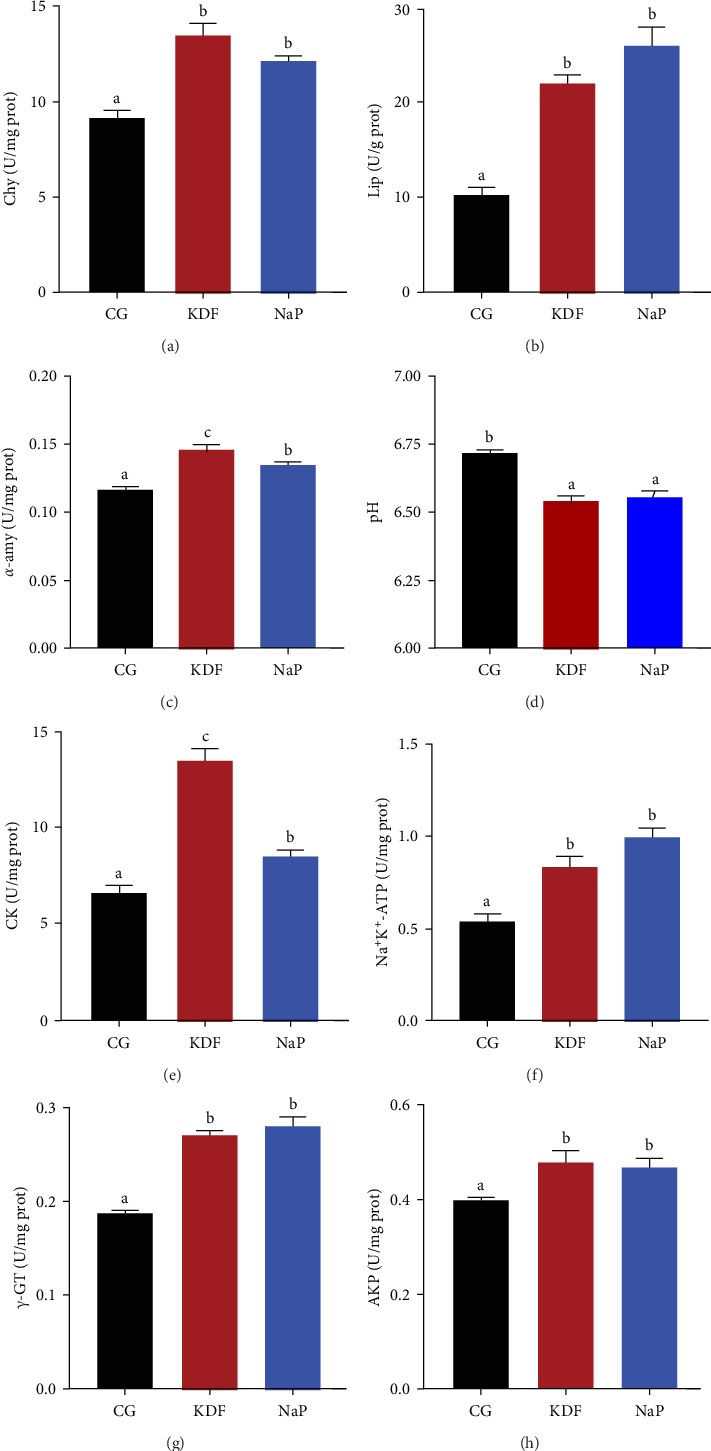
Intestinal enzymes of *T. ovatus* fed the experimental diets. (a) Chy, Chymotrypsin. (b) Lip, Lipase. (c) α-amy, α-amylase. (d) pH, potential of Hydrogen. (e) CK, creatine kinase. (f) Na^+^K^+^-ATP, Na^+^ K^+^ ATPase. (g) γ-GT, γ-glutamyl transferase. (h) AKP, alkaline phosphatase. Values represent the mean ± SEM (*n* = 5). Different letters above the bar represent significant differences among different groups (*p* < 0.05).

**Figure 2:**
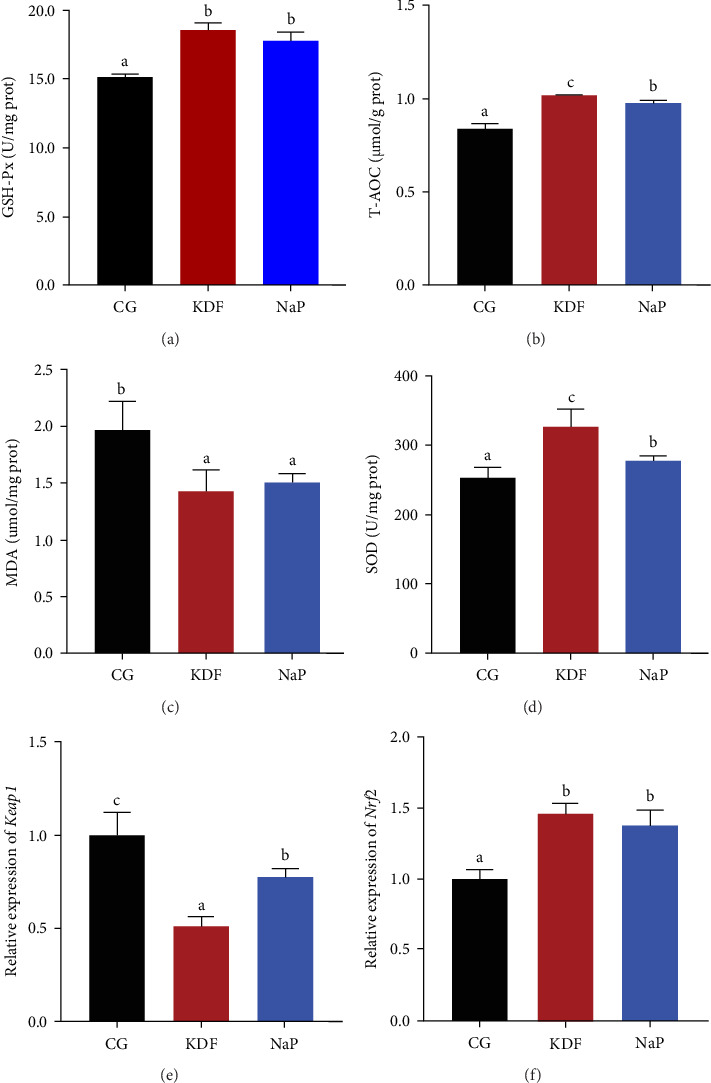
Intestinal antioxidant function of *T. ovatus* fed the experimental diets. (a) GSH-Px, glutathione peroxidase. (b) T-AOC, total antioxidant capacity. (c) SOD, superoxide dismutase. (d) MDA, malondialdehyde. (e) Keap1, Kelch-like ECH-associated protein 1. (f) Nrf2, Nuclear factor erythroid 2-related factor 2. Values represent the mean ± SEM (*n* = 5). Different letters above the bar represent significant differences among different groups (*p* < 0.05).

Data presented in Figure 1 demonstrated that compared to the CG group, activities of Chy, Lip, α-amy, CK, Na^+^K^+^-ATP, γ-GT, and AKP in the KDF and NaP groups significantly increased (*p* < 0.05), while the pH value pronouncedly decreased (*p* < 0.05). In addition, α-amy and CK in the KDF group had significantly higher activities than those in the NaP group (*p* < 0.05). As indicated in Figure 2, GSH-Px and SOD activities in the KDF and NaP groups were markedly higher relative to the CG group (*p* < 0.05). The supplementation of KDF and NaP in diets notably increased T-AOC content and remarkably decreased MDA level (*p* < 0.05). Compared with that in the CG group, the expression of intestinal *Nrf2* gene was significantly upregulated but the expression of *Keap1* gene significantly downregulated in the KDF and NaP groups (*p* < 0.05).

### 3.2. Gut Microbiota

**Figure 3:**
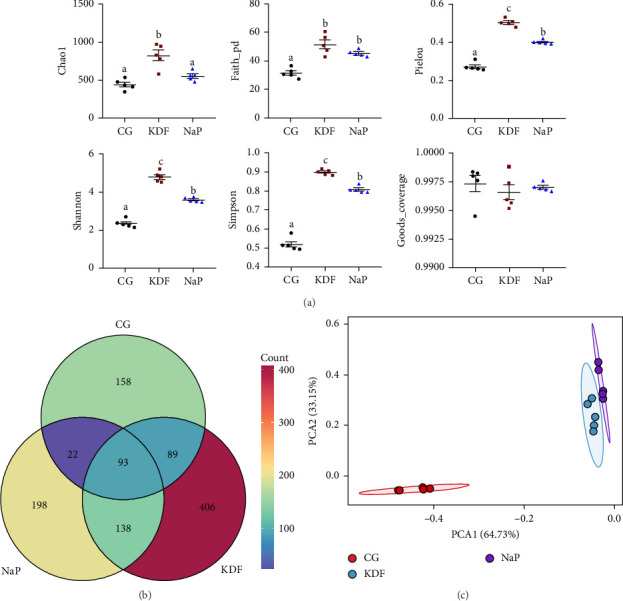
The diversity parameters of intestinal microbiota of *T. ovatus* fed the experimental diets. (a) Alpha diversity, (b) venn diagram, and (c) beta diversity of intestinal microbiota based on principal component analysis (PCA). Values represent the mean ± SEM (*n* = 5). Different lowercase letters above the scatter represent significant differences among different groups (*p* < 0.05).

To further elucidate the impact of adding KDF and NaP on gut health, we investigated alterations in the structure of the intestinal microbiota. As shown in Figure 3a, in terms of intestinal alpha diversity indices, the inclusion of KDF and NaP in the diets significantly increased Faith_pd, Pielou, Simpson, and Shannon compared to the CG group (*p* < 0.05). Venn diagram analyzed the similarity and difference of OTUs among the three groups (Figure 3b). There were 1104 OTUs in the CG, KDF, and NaP groups and these groups exhibited 93 identical OTUs in their collective microbiome profiles. In addition, 158, 406, and 198 OTUs were uniquely identified in the CG, KDF, and NaP groups, respectively. Beta diversity was analyzed by using principal component analysis (PCA) based on Euclidean distance (Figure 3c). The clustering outcomes showed a clear distinction in the KDF group and NaP group compared to the CG group, indicated adding KDF and NaP obviously altered the diversity of intestinal microbiota.

**Figure 4:**
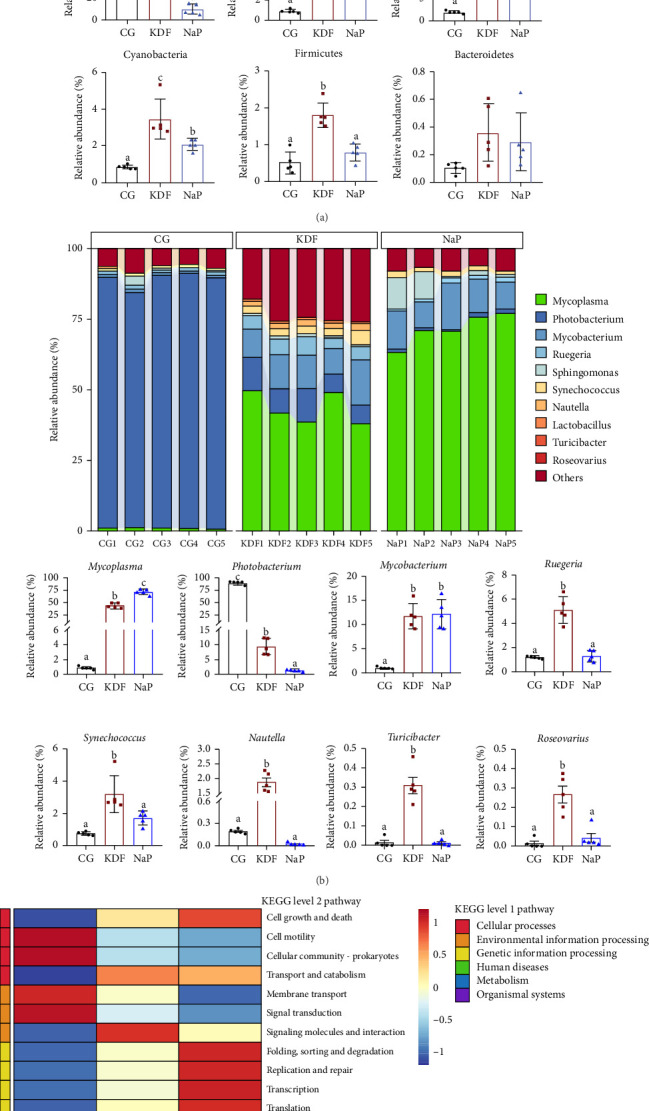
Intestinal microbiota composition and functional prediction of *T. ovatus* fed three diets. (a) Bacteria composition at phylum level (b) Bacteria composition at genus level. (c) Functional prediction of microbiota. Values represent the mean ± SEM (*n* = 5). Different lowercase letters above the scatter represent significant differences among different groups (*p* < 0.05).

The intestinal microbiota architecture was characterized at the phylum and genus levels (Figure 4a,b). At the phylum level, the five most abundant phyla across all three groups were consistently identified as Proteobacteria, Tenericutes, Actinobacteria, Cyanobacteria, and Firmicutes, which constituted 99.8%, 99.2%, and 98.9% of all bacterial phyla, respectively. In terms of the relative abundance, Proteobacteria obviously decreased in KDF and NaP groups while Tenericutes, Actinobacteria, and Cyanobacteria obviously increased compared to the CG group (*p* < 0.05). The relative abundance of Firmicutes significantly increased in the KDF group compared to the CG group (*p* < 0.05), while no significant difference was observed in the NaP group. The inclusion of KDF and NaP in the diets did not lead to any obvious difference in the relative abundance of Bacteroidetes (*p* > 0.05). At the genus level, the composition of the bacterial community exhibited significant alterations in the KDF and NaP groups compared to the CG group. After feeding the diets supplemented with KDF and NaP, a significant decline in the relative abundance of *Photobacterium* and significant increase of *Mycoplasma* and *Mycobacterium* were detected (*p* < 0.05). In addition, the relative abundance of *Ruegeria*, *Synechococcus*, *Nautella*, *Turicibacter*, and *Roseovarius* in the KDF group obviously increased in comparison with the CG group (*p* < 0.05), while these microorganisms were not difference between CG and NaP groups (*p* > 0.05). As indicated in Figure 4c, function prediction of the gut microbial community was estimated using PICRUSt2. At KEGG level 1, six KEGG primary metabolic pathways were annotated in the three groups. Dietary KDF and NaP obviously changed the function of intestinal microbiota at KEGG level 2 pathway. In particular, the metabolic pathways, such as “Cell growth and death,” “Transport and catabolism,” “Amino acid metabolism,” “Biosynthesis of other secondary metabolites,” “Carbohydrate metabolism,” “Energy metabolism,” “Lipid metabolism,” “Metabolism of cofactors and vitamins,” “Digestive system,” and “Immune system” in the KDF and NaP groups had higher enrichment than those in the CG group.

### 3.3. Intestinal SCFAs Level

**Figure 5:**
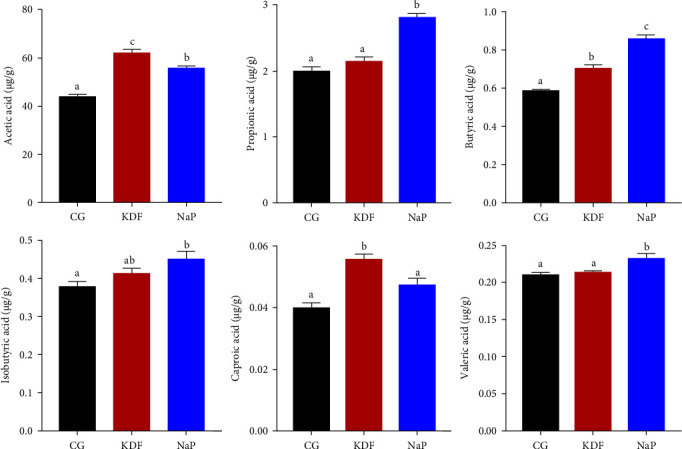
Intestinal SCFAs contents of *T. ovatus* fed three diets. Values represent the mean ± SEM (*n* = 5). Different lowercase letters above the bar represent significant differences among groups (*p* < 0.05).

To explore the impact of KDF and NaP on intestinal SCFAs levels, all SCFAs were subjected to supervised OPLS-DA (Figure S1). Samples from the three groups showed clear separation in the OPLS-DA model, suggesting diets supplementation with KDF and NaP significantly influenced intestinal SCFAs levels. Bar chart depicted the relative abundance of intestinal SCFAs among the three groups (Figure 5). In comparison with the CG group, the levels of acetic acid and butyric acid in the KDF and NaP group significantly increased (*p* < 0.05). Compared to the CG group, the levels of propionic acid, isobutyric acid, and valeric acid significantly increased in NaP group (*p* < 0.05), while caproic acid exhibited a significant increase in KDF group (*p* < 0.05).

### 3.4. Intestinal Lipidomic Variations

**Figure 6:**
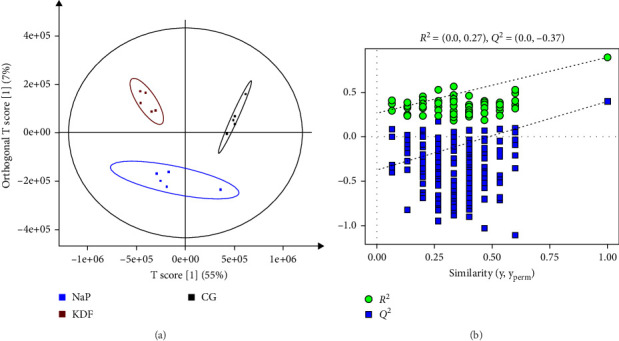
Multivariate statistical analysis based on OPLS-DA of lipid metabolites in the intestinal contents of *T. ovatus*-fed three diets. (a) OPLS-DA score plot. (b) Permutation test plot.

**Figure 7:**
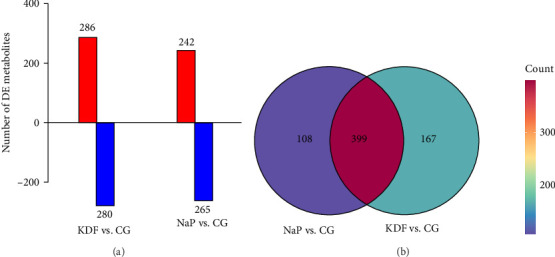
The differential lipids identified in the intestinal contents of *T. ovatus*-fed three diets. (a) The number of differential lipids identified in different groups. (b) Venn analysis of differential lipids among the different groups.

A total of 4985 lipid molecules were identified, belonging to 68 lipid classes. The major lipid class was triglyceride (TG, 43.83%), followed by phosphatidylcholine (PC, 16.09%), ceramide (Cer, 9.26%), diglyceride (DG, 8.15%), and methyl PC (MePC, 5.37%) (Figure S2). As reveled by the OPLS-DA score plots and the random permutations plot, the separations were observed between the three groups, suggesting diets supplementation with KDF and NaP significantly influenced intestinal lipids molecules levels (Figure 6). To further analyze lipidomic data, the methods (*p* < 0.05, VIP > 1 and fold change >1.5 or <0.05) were used to detect the differential lipid species. The results showed there were 566 differential lipid species were identified between CG and KDF group, 507 differential lipid species between CG and NaP group (Figure 7a). Compared with the CG group, 286 elevated and 280 reduced lipids were identified in the KDF group, while 242 elevated and 265 reduced lipids in the NaP group. Venn diagram showed 399 lipids were common to KDF and NaP groups, with uniquely identified lipids numbering 167 in KDF and 108 in NaP (Figure 7b). TG predominated among co-occurring lipid classes, succeeded by PC, MePC, DG, and Cer. The top five lipid classes unique to KDF group were TG, PC, DG, MePC, and HexCer, while those in the NaP group were TG, DG, PC, Cer, and HexCer (Table S3).

**Figure 8:**
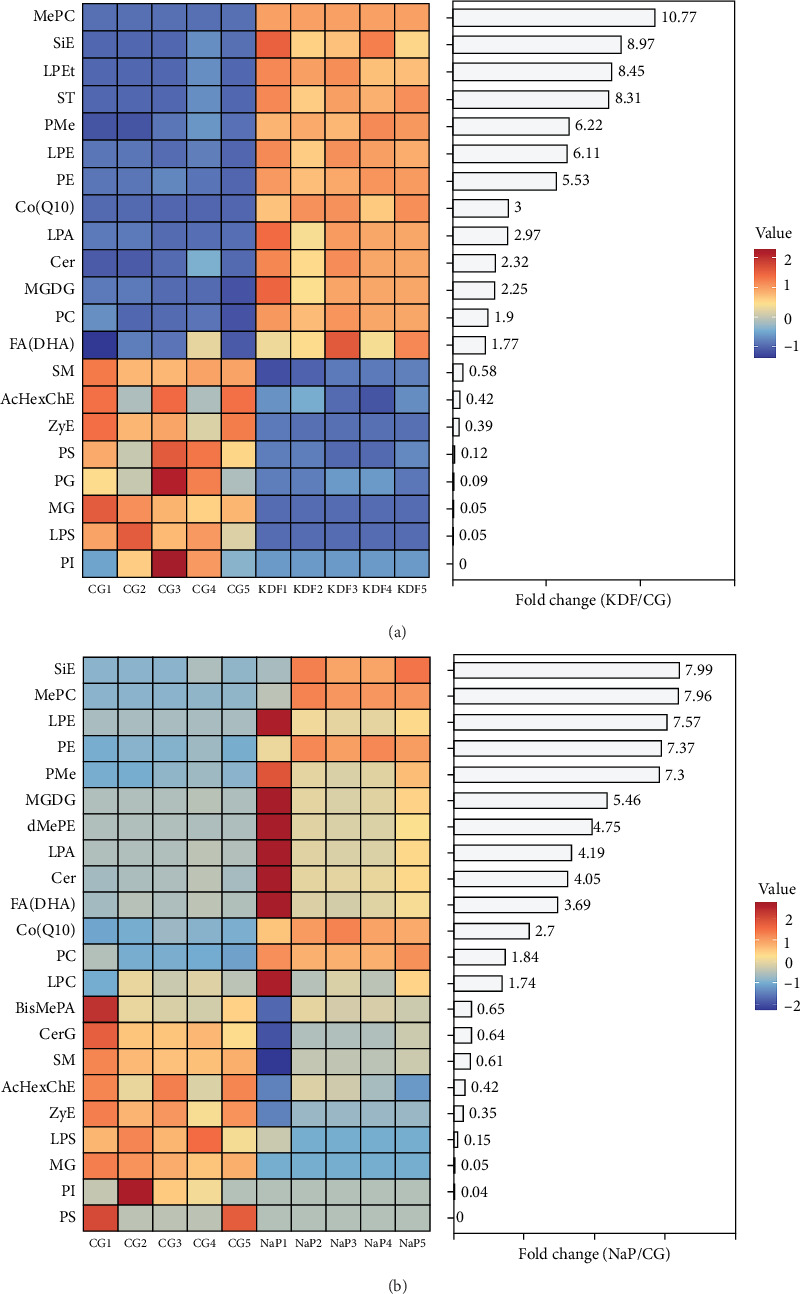
Abundance and fold change of the differential lipid classes in the intestinal contents of *T. ovatus*-fed three diets. (a) The comparison group of KDF vs. CG. (b) The comparison group of NaP vs. CG. (AcHexChE, acetylated glucosyl cholesterol ester; BisMePA, bis-methyl phosphatidic acid; Cer, ceramide; CerG, glucocerebroside; Co, coenzyme; dMePE, dimethylphosphatidylethanolamine; FA, fatty acid; LPA, lysophosphatidic acid; LPC, lysophosphatidylcholine; LPE, lysophosphatidylethanolamine; LPEt, lysophosphatidylethanol; LPS, lysophosphatidylserine; MePC, methyl phosphatidylcholine; MG, monoglyceride; MGDG, monogalactosyldiacylglycerol; PC, phosphatidylcholine; PE, phosphatidylethanolamine; PG, phosphatidylglycerol; PI, phosphatidylinositol; PMe, phosphatidylmethanol; PS, phosphatidylserine; SiE, sitosteryl; SM, sphingomyelin; ST, sulfatidate; ZyE, zymosterol).

The alteration of differential lipids at the lipid class level were analyzed. These differential lipid species were divided into 21 lipid classes between CG and KDF group, and 22 lipid classes between CG and NaP group (Figure 8). Compared with CG group, the abundance of PC, PE, MePC, PMe, LPE, LPA, Cer, SiE, MGDG, Co, and FA were increased, while PI, PS, LPS, SM, MG, ZyE, and AcHexChE were decreased in the KDF and NaP group; ST and LPEt were only increased and PG only declined in the KDF group, while LPC and deMePE were only augmented and BisMePA and CerG only decreased in the in the NaP group.

### 3.5. Correlation Analysis Integrating Gut Microbiota, Physiological Indices, and Lipid Metabolites

**Figure 9:**
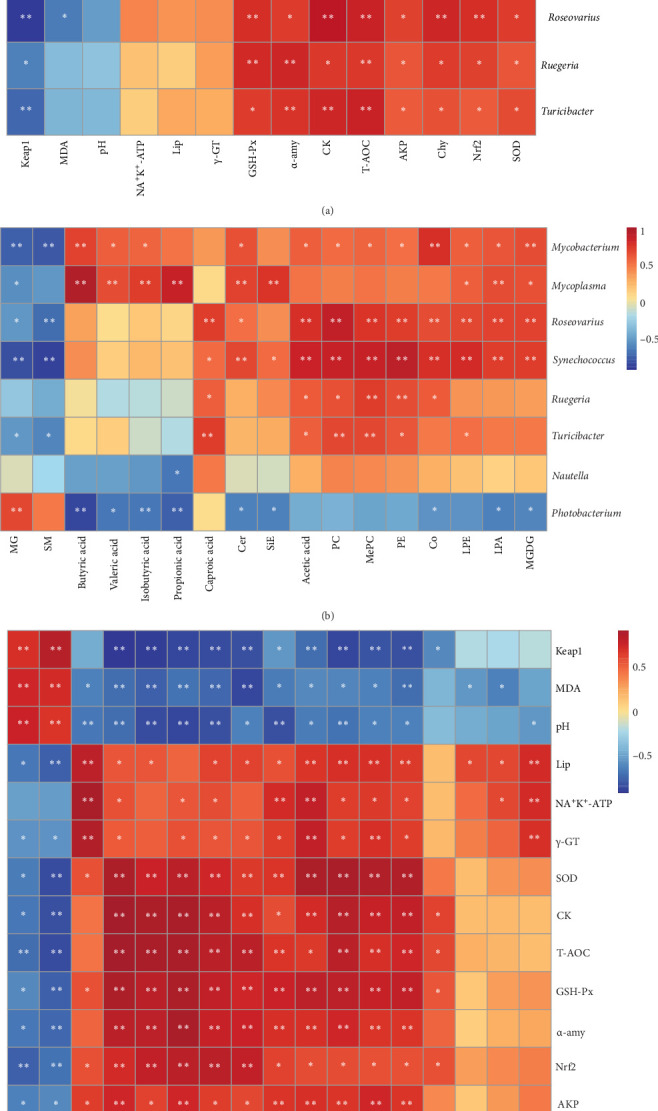
Spearman's correlation analysis. (a) Correlation heatmap analysis between intestinal microbiota at the genus level and physiologic indices. (b) Correlation heatmap analysis between the genus-level microbiota and lipid metabolites. (c) Correlation heatmap analysis between physiologic indices and lipid metabolites. The asterisks *⁣*^*∗*^ and *⁣*^*∗∗*^, respectively, mean *p* < 0.05 and *p* < 0.01.

The results showed that *Photobacterium* had negative correlations with physiological indices, such as Na^+^K^+^-ATP, Lip, γ-GT, Chy, AKP, and *Nrf2*, and positive correlations with MDA and pH (*p* < 0.05). *Mycobacterium*, *Syncchococcus*, *Roseovarius*, *Ruegeria*, and *Turicibacter* had negative correlations with *Keap1* while positive correlations with GSH-Px, α-amy, CK, T-AOC, AKP, Chy, *Nrf2*, and SOD (*p* < 0.05) (Figure 9a). In addition, *Photobacterium* were negatively correlated with lipid molecules, such as MGDG, LPA, Co, SiE, Cer, propionic acid, isobutyric acid, valeric acid and butyric acid, and positively correlated with MG (*p* < 0.05). *Mycobacterium*, *Roseovarius* and *Syncchococcus* exhibited positive correlations with acetic acid, PC, MePC, PE, Co, LPE, LPA, and MGDG, while showing negative correlations with MG and SM (*p* < 0.05) (Figure 9b). Analysis of statistical association demonstrated that SOD, CK, T-AOC, GSH-Px, α-amy, *Nrf2*, AKP, and Chy manifested negative correlated with MG and SM, exhibiting concurrent positive correlations with PC, MePC, PE, acetic acid, Co, SiE, Cer, LPE, LPA, and MGDG (*p* < 0.05). On the contrary, *Keap1*, MDA, and pH were positively correlated with MG and SM, while negatively correlated with PC, MePC, PE, acetic acid, Co, SiE, Cer, LPE, LPA, and MGDG (*p* < 0.05) (Figure 9c).

## 4. Discussions

The gut is a critical organ in fish, responsible for digestion, nutrient absorption, metabolic processing, and immune defense. The capacity of fish to effectively assimilate dietary nutrients is predominantly influenced by the intestinal enzymatic activities [38]. Gut-associated digestive enzymes, such as Chy, Lip, and amylase, are primarily involved in the enzymatic breakdown of proteins, lipids, and carbohydrates, respectively [39]. The outcomes of this research revealed that KDF and NaP markedly enhanced the AMS, Lip, and Chy activities of *T. ovatus*, indicating that the addition of KDF and NaP contributed to the promotion of digestive enzyme activity in the intestines. The brush-border enzymes, including Na^+^K^+^-ATP, AKP, γ-GT, and CK, play key roles in nutrient absorption. They facilitate the associated transport processes, including ion gradients, phosphate hydrolysis, peptide transport, and cellular energy provision, enabling efficient uptake of dietary components [40–42]. In this study, the addition of KDF and NaP pronouncedly elevated the activities of gut brush-border enzymes, which revealed that KDF and NaP enhanced the processes of nutrients uptake, transport and metabolism of *T. ovatus*. Recent studies reported that dietary KDF and NaP addition improved the enzymes activities associated with intestinal digestion and absorption in juvenile grass carp [25] and koi carp (*Cyprinus carpio* koi) [43], which were consistent with our result. In addition, the gut pH significantly decreased in the fish-fed KDF and NaP diets. Research indicated that the organic acids could reduce intraluminal gut pH and promote secretin secretion, which potentiates enzymatic activity of the pancreas [12, 26]. These findings demonstrated that KDF and NaP could improve digestion and absorption capacity of fish through reducing intestinal pH value.

Beyond facilitating the nutrient metabolism, the gut serves as a key barrier protecting animals from external stressors. In general, exogenous challenges will trigger the overproduction of reactive oxygen species (ROS), causing the generation of MDA and intestinal oxidative damage [44]. The intestinal antioxidant system contributes significantly in maintaining intestinal health [45]. SOD, GSH-Px, and T-AOC are widely recognized as essential biomarkers of antioxidant status, which are involved in ROS decomposition [44]. This study indicated that dietary KDF and NaP resulted in higher intestinal antioxidant enzyme activities and lower MDA levels, suggesting that KDF and NaP enhanced intestinal antioxidant capacity. Additionally, dietary SCFAs suppressed *Keap1* transcription and enhanced *Nrf2* expression. The Keap1/Nrf2 pathway is the predominant signaling cascade governing expression of antioxidant genes [46]. Generally, *Keap1* mediates the inhibition of *Nrf2* by facilitating its association into a molecular complex [47]. *Nrf2*, as a master transcriptional regulator, binds antioxidant response elements, and activates the genes encoding antioxidative enzyme, thereby mitigating oxidative damage [48]. Evidence from our experimental data underscores KDF and NaP enhanced the antioxidant function of *T. ovatus* by activating the Keap1/Nrf2 pathway.

The shaping of host phenotypes (e.g., growth, metabolism, and immunity) was modulated by gut microorganisms [49]. Gut health is closely linked to the modulation of the microbiota through dietary interventions [50]. In this study, alpha diversity metrics (Simpson, Shannon, Pielou, and Faith) exhibited marked elevations in the KDF and NaP group relative to CG. What's more, dietary KDF and NaP inclusion significant altered the beta diversity of *T. ovatus*. Research indicates that the gut microflora diversity in fish is significantly influenced by dietary habits and nutritional profiles [6]. Earlier findings have confirmed that dietary sodium acetate restructured intestinal microbial structure of *T. ovatus* and *Micropterus salmoides* [33, 35]. A recent study found that NaP could significantly improve the diversity indexes of tilapia [51]. These findings supported our studies, indicating supplemented KDF and NaP promoted diversity of *T. ovatus*.

In this study, Proteobacteria, Tenericutes, Actinobacteria, Cyanobacteria, and Firmicutes comprised the core microbiota in CG, KDF and NaP groups with total abundances of 99.8 %, 99.2 %, and 98.9 %, respectively. The present study demonstrated marked decline in phylum Proteobacteria and genus *Photobacterium*, and significant increase in phyla Tenericutes and Actinobacteria, as well as genera *Mycoplasma* and *Mycobacterium* following the administration of KDF and NaP. Proteobacteria and *Photobacterium* are regarded as main pathogenic bacteria of *T. ovatus* enteritis [52]. Tenericutes is thought to be advantageous for fish health by competitively excluding pathogenic bacteria [53]. *Mycoplasma* belongs to Tenericutes primarily synthesizes lactate and acetate as dominant fermentation products [54], which are beneficial for fish health. Actinobacteria are widely known to produce antibacterial and enhance host immune function [55, 56]. Findings found immunomodulation by certain *Mycobacterium* strains confers immunological benefits to salmonids in the absence of negative outcomes [57]. In addition, the abundance of potential beneficial bacteria, such as phyla Cyanobacteria and Firmicutes significantly increased in the KDF and NaP groups. These findings suggested dietary KDF and NaP inclusion positively regulate the gut microbiota. Dietary KDF and NaP inclusion obviously modified the abundance ratios of core phyla without disrupting their dominance profile, suggesting a functional reorganization in gut microbial physiology. The subsequent functional prediction in this study also verified that adding KDF and NaP drived functional remodeling specifically in “Cellular processes,” “Environmental Information Processing,” “Genetic Information Processing,” “Human Diseases,” “Metabolism,” and “Organismal Systems” of intestinal microbiota.

Being the principal energy provider for intestinal epithelial cells, SCFAs contribute significantly to lipid metabolism regulation and maintenance of intestinal function in animals [58]. This study revealed elevated concentrations of intestinal SCFAs, with the greatest increase observed for acetic acid and butyric acid. Microbial fermentation serves as the principal source of SCFA, with variations in their abundance and speciation directly dependent on the prevalence of SCFA-synthesizing bacteria [59]. This is consistent with our results mentioned above regarding the significant increase in Actinobacteria, Firmicutes and Bacteroidetes. These findings suggest that KDF and NaP can elevate the levels of intestinal SCFAs by regulating the abundance of microbiota, thus improving the gut health of *T. ovatus*.

Lipid metabolites produced by the microorganisms are key components of their physiological effects. These metabolites function as molecular mediators transmitting regulatory signals from gut microbiota to host cells, modulating developmental trajectories, physiological homeostasis, and disease mechanisms in the host [60]. The present study demonstrated dietary KDF and NaP modified the lipid profiles to varying extents. Integral to the phospholipid bilayer structure, PC, PE, and their derivative underpin cellular membrane integrity by mediating core physiological functions—energy metabolism, cell signaling, structural support, and lipid transport [61]. A recent study has demonstrated that PC effectively suppresses pro-inflammatory mediators, and concurrently heightened synthesis of proteins critical for epithelial junctional complexes [62]. Decreased PE levels correlate with reduced mucus layer thickness, triggering epithelial barrier failure and inflammatory cascades in the gut [63]. LPA alleviates intestinal inflammation and maintains gut homeostasis through activation of the G protein-coupled receptor 35 (GPR35) signaling pathway in colonic macrophages [64]. A recent study demonstrated Cer potentiates extreme environmental adaptation in nematodes via p38/MAPK-dependent cytoprotection and microbiome-derived resilience mechanisms, attenuating oxidative stress-induced senescence [65]. Increase SM expression might induced pro-inflammatory effect [66]. SiE exerts its effect on antioxidant enzymes through modulating the estrogen receptor/PI3K pathway. It also modulated glutathione homeostasis, implying functionality as a potent free radical scavenger [67]. Significant increase in PC, PE, MePC, LPA, Cer, and SiE, and decrease in SM were observed of the KDF and NaP groups, which implied that adding KDF and NaP into fish diets affected the membrane homeostasis of the intestine and improved intestinal antioxidant and immune function of *T. ovatus*. MGDGs can reduce the levels of TG and FFA in adipocytes [68]. LPE emulsifies lipids, facilitating the nutrient bioavailability [69]. Coenzyme Q10 is an essential intracellular coenzyme that possesses significant antioxidant and immunoenhancing properties [70]. As a crucial fatty acid, DHA significantly improves intestinal development, metabolic processes, and immune homeostasis in fish [71, 72]. Increased MGDG, LPE, Co, and decreased MG levels in this study suggested adding KDF and NaP was beneficial for lipid metabolism improvement.

Diet, gut microbiota, and metabolic function of host are closely linked and interact with each other. Prior researches have confirmed that changes of intestinal microbiota can affect intestinal digestive and immune function in fish [73–76]. In the present study, *Photobacterium*, *Mycobacterium*, *Mycoplasma*, *Syncchococcus*, *Roseovarius*, *Ruegeria*, and *Turicibacter* showed significant correlations with digestive and antioxidative functions, which may be driven by compositional perturbations within key intestinal bacterial communities. Furthermore, *Mycobacterium*, *Mycoplasma*, *Syncchococcus*, *Roseovarius*, *Ruegeria*, and *Turicibacter* had consistent positive or negative associations with various lipid metabolites, suggesting a potential for functional synergy in their metabolic roles. However, the association patterns of *Photobacterium* with these metabolites were distinct from those observed in the aforementioned bacteria, indicating that there is a degree of antagonism between *Photobacterium* and aforementioned bacteria. In addition, correlation analysis between physiological indices and lipid molecules revealed that digestive and antioxidative functions of *T. ovatus* were influenced by lipid molecules levels. These results suggested dietary supplementation with KDF and NaP may improve the digestive and immune functions in *T. ovatus* through a complex interaction-mediated mechanism between the gut microbiota and intestinal metabolites.

## 5. Conclusion

This study revealed the effects of dietary KDF and NaP on the intestinal health of *T. ovatus*. KDF and NaP supplementation enhanced activities of digestive and antioxidative enzymes, decreased pH value as well as regulating the expression level of *Keap1* and *Nrf2*. Furthermore, dietary KDF and NaP promoted gut microbial architecture by upregulating diversity, worsening the pathogens colonization, and promoting the prevalence of beneficial bacteria, meanwhile increased the SCFAs level and regulated the lipid profile of intestinal content, which might be beneficial for the improvement of digestive and antioxidative functions. In summary, dietary KDF and NaP inclusion may regulate the gut microbiota and lipid profile to influence the intestinal metabolism and immune response, thus improving the intestinal health of *T. ovatus*.

## Data Availability

The data that support the findings of this study are available from the corresponding author upon reasonable request.

## Supporting Information

Supporting Information The supporting information include the formulation and proximate analysis of the experimental diets (Table S1), the information of targeted gene primer sequences (Table S2), the co-owned and unique lipid amounts between the different groups (Table S3), multivariate statistical analysis based on OPLS-DA of SCFAs level (Figure S1), and the classification of the analyzed lipids (Figure S2).
