# Supplementary material for: Potential Mechanisms of Dietary Potassium Diformate and Sodium Propionate Driving Intestinal Microbiota and Lipid Metabolites to Modulate Intestinal Health of Trachinotus ovatus
Source: Aquac Nutr. 2025 Nov 18;2025:5594216. doi: 10.1155/anu/5594216 (PMC12646738; doi:10.1155/anu/5594216)
Supplement: Supporting Information — The supporting information include the formulation and proximate analysis of the experimental diets (Table S1), the information of targeted gene primer sequences (Table S2), the co-owned and unique lipid amounts between the different groups (Table S3), multivariate statistical analysis based on OPLS-DA of SCFAs level (Figure S1), and the classification of the analyzed lipids (Figure S2). [file 5594216.f1.docx]

**TABLE S1** Formulation and proximate analysis of the experimental diets (on dry weight basis, %).

| Ingredients^*^ | CG | KDF | NaP |
| --- | --- | --- | --- |
| Fish meal | 25.00 | 25.00 | 25.00 |
| Soybean meal | 15.00 | 15.00 | 15.00 |
| Soy protein concentrate | 10.00 | 10.00 | 10.00 |
| Peanut meal | 10.00 | 10.00 | 10.00 |
| Pork powder | 6.00 | 6.00 | 6.00 |
| Brewers dried yeast | 5.00 | 5.00 | 5.00 |
| Wheat flour | 18.0 | 18.0 | 18.0 |
| Fish oil | 6.00 | 6.00 | 6.00 |
| Soybean Lecithin | 1.00 | 1.00 | 1.00 |
| Vitamin and mineral mix^1^ | 1.00 | 1.00 | 1.00 |
| Choline chloride | 0.50 | 0.50 | 0.50 |
| Ca(H_2_PO_4_)_2_ | 0.50 | 0.50 | 0.50 |
| L-methionine | 0.20 | 0.20 | 0.20 |
| Antioxidant | 0.10 | 0.10 | 0.10 |
| Attractant | 0.50 | 0.50 | 0.50 |
| Potassium diformate | 0 | 0.66 | 0 |
| Sodium propionate | 0 | 0 | 0.60 |
| Zeolite powder | 1.20 | 0.54 | 0.60 |
| Proximate composition (%) | |  |  |
| Moisture | 9.56 | 9.37 | 9.33 |
| Crude protein | 44.27 | 44.31 | 44.12 |
| Crude lipid | 10.77 | 10.58 | 10.60 |
| Ash | 9.63 | 9.39 | 9.24 |

^*^Fish meal, soybean meal, peanut meal, wheat flour, soybean lecithin, vitamin and mineral mix, choline chloride, monocalcium phosphate, attractant, zeolite powder were purchased by Kingkey Smart Agri Technology Co., Ltd. (Shenzhen, China). Soy protein concentrate, brewers dried yeast, antioxidant were purchased by Guangdong Yuequn Ocean Biological Research Development Co., Ltd. (Jieyang, China). Pork powder weas purchased by Guangdong Xunyuan Nutrition Technology Co., Ltd. (Jieyang, China). Fish oil was purchased by Yongxing Concentrated Feed Co., Ltd. (Guangzhou, China). L-methionine was purchased by Changyi Pharmaceutical Co., Ltd. (Zhejiang, China).

^1^Vitamin and mineral mix provided the following per kg of diet: vitamin A (5,00,000 IU) 40 mg, vitamin B_1_ 40 mg, vitamin B_2_ 93.75 mg, vitamin B_6_ 20 mg, vitamin B_12_ (1%) 45 mg, vitamin K_3_ (50%) 300 mg, inositol 400 mg, calcium pantothenate 250 mg, nicotinic acid 450 mg, folic acid 6 mg, biotin (2%) 10 mg, vitamin D_3_ (5,00,000 IU) 15 mg, vitamin E (50%) 300 mg, unite bran 2990.25 mg, NaF 4 mg, KI 1.6 mg, CuSO_4_•5H_2_O 20 mg, CoCl_2_·6H_2_O (1%) 100 mg, FeSO_4_•H_2_O 160 mg, ZnSO_4_•H_2_O 100 mg, MnSO_4_•H_2_O 120 mg, MgSO_4_•7H_2_O 2.4 g, Ca(H_2_PO_4_)_2_•H_2_O 6.0 g, NaCl 200 mg, zeolite powder 30.90 g.

**TABLE S2** Targeted gene primer sequences used for qRT-PCR analysis

| Genes | | Sequence | Length (bp) | TM (℃) | Sources |
| --- | --- | --- | --- | --- | --- |
| Keap1 | F | AGAGGATGGAGATGGCACAG | 20 | 57.45 | Zhou et al., 2020 |
|  | R | CATTGGTTGGTCTTGGGATT | 20 | 53.35 |  |
| Nrf2 | F | AGCTTGGCCTTCATCAAAT | 19 | 50.85 | Zhou et al., 2020 |
|  | R | GAGTATGGCTGTCCTTCTTCA | 21 | 55.61 |  |
| β-Actin | F | TACGAGCTGCCTGACGGACA | 20 | 59.50 | Tan et al., 2017 |
|  | R | GGCTGTGATCTCCTTCTGC | 19 | 57.32 |  |

Zhou C., Lin H., Huang, Z., et al. Effects of dietary leucine levels on intestinal antioxidant status and immune response for juvenile golden pompano (*Trachinotus ovatus*) involved in Nrf2 and NF-κB signaling pathway. Fish & Shellfish Immunology, 2020, 107, 336–345,

Tan X., Sun Z., Huang et al. Effects of dietary hawthorn extract on growth performance, immune responses, growth- and immune-related genes expression of juvenile golden pompano (*Trachinotus ovatus*) and its susceptibility to *Vibrio harveyi* infection. Fish & Shellfish Immunology, 2017, 656–664.

**TABLE S3** The co-owned and unique lipid amounts in the intestinal contents of *T. ovatus* between the different groups.

| Lipids class | Co-owned in KDF and NaP | Unique in KDF | Unique in NaP |
| --- | --- | --- | --- |
| Glycerophospholipids (GPLs) |  |  |  |
| Phosphatidylcholine (PC) | 56 | 42 | 14 |
| Phosphatidylethanolamine (PE) | 5 | 3 | 1 |
| Phosphatidylglycerol (PG) | 1 | 0 | 1 |
| Phosphatidylserine (PS) | 1 | 2 | 0 |
| Phosphatidylinositol (PI) | 1 | 0 | 1 |
| Lyso-phosphatidylcholine (LPC) | 3 | 3 | 2 |
| Lyso-phosphatidylethanolamine (LPE) | 6 | 0 | 0 |
| Lyso-phosphatidylserine (LPS) | 1 | 0 | 1 |
| BisMePA | 9 | 2 | 3 |
| LdMePE | 0 | 3 | 0 |
| MePC | 39 | 18 | 3 |
| LBPA | 1 | 0 | 0 |
| PMe | 1 | 0 | 0 |
| LPEt | 0 | 2 | 0 |
| dMePE | 0 | 0 | 1 |
|  |  |  |  |
| Sphingolipids (SLs) | | | |
| Sphingomyelin (SM) | 6 | 7 | 1 |
| Ceramides (Cer) | 13 | 2 | 5 |
| CerG |  |  |  |
| ST | 0 | 1 | 0 |
| HexCer | 13 | 9 | 4 |
|  |  |  |  |
| Glycerolipids (GLs) | | | |
| Monoglyceride (MG) | 1 | 0 | 0 |
| Diglyceride (DG) | 36 | 18 | 16 |
| Triglyceride (TG) | 193 | 54 | 51 |
|  |  |  |  |
| Sterols | | | |
| ZyE | 5 | 0 | 0 |
| SiE | 1 | 0 | 3 |
|  |  |  |  |
| Other lipids |  |  |  |
| FA | 1 | 0 | 0 |
| Co | 1 | 0 | 0 |
| AcHexChE | 1 | 0 | 0 |
| MGDG | 4 | 1 | 1 |


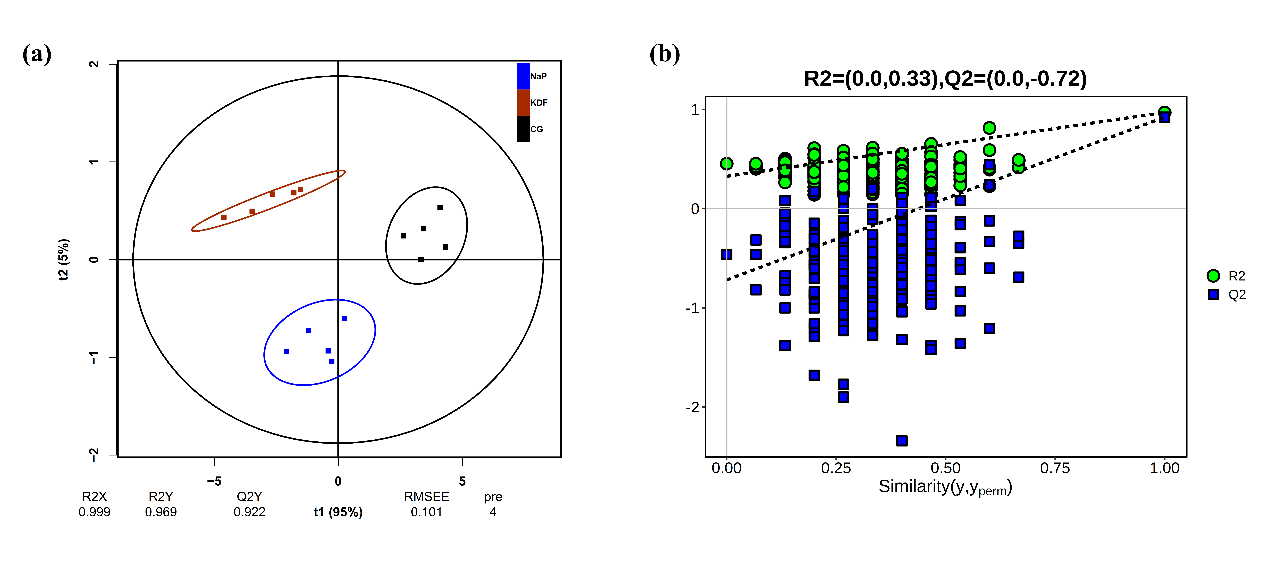


**FIGURE S1.** Multivariate statistical analysis based on OPLS-DA of SCFAs level in the intestinal contents of *T. ovatus* fed three diets. (a) OPLS-DA score plot. (b) Permutation test plot.


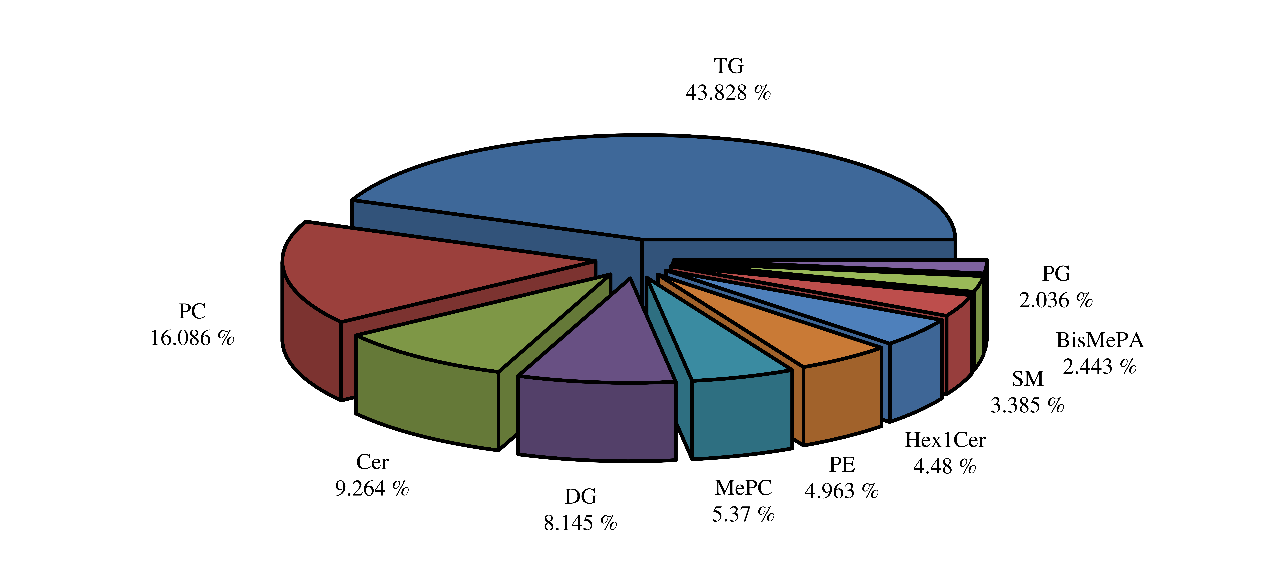


**FIGURE S2.** The classification of the analyzed lipids from the intestinal contents of *T. ovatus*
